# Use of a food neophobia test to characterize personality traits of dairy calves

**DOI:** 10.1038/s41598-020-63930-8

**Published:** 2020-04-28

**Authors:** Joao H. C. Costa, Heather W. Neave, Daniel M. Weary, Marina A. G. von Keyserlingk

**Affiliations:** 1 Animal Welfare Program, Faculty of Land and Food Systems, University of British Columbia, 2357 Main Mall, Vancouver, B.C. V6T 1Z4 Canada; 2 Present Address: Dairy Science Program, Department of Animal and Food Sciences, University of Kentucky, 325 Cooper Dr., Lexington, 40546 USA; 3 Present Address: AgResearch Ltd., Ruakura Research Centre, 10 Bisley Rd, Hamilton, 3214 New Zealand

**Keywords:** Behavioural methods, Animal behaviour

## Abstract

Food neophobia, i.e. the avoidance of novel foods, is common in ruminants and may provide a biologically relevant and practical way to test individual responses to novelty or challenge. We aimed to determine if behavioural responses in a food neophobia test (exposure to a novel total mixed ration) reflected boldness and exploratory personality traits derived from 3 traditional tests (open field, novel human and novel object) in dairy calves. We performed two Principal Component Analyses, one using behaviours from 3 traditional tests (3 factors: ‘Bold’, ‘Exploratory’ and ‘Active’), and one using behaviours from the food neophobia test (3 factors: ‘Eating’, ‘Inspecting’, and ‘Avoidance’). A regression analysis determined if individual factor scores from the food neophobia test predicted factor scores from the traditional tests. Contrary to our expectations, ‘Avoidance’ (latencies to approach and eat the novel food) did not predict boldness trait, and the factors ‘Inspecting’ (time spent inspecting food and empty buckets) and ‘Eating’ (time spent eating food and total intake) did not predict exploration trait, but they did predict active trait. These results suggest that the food neophobia test in our study resulted in context-specific behaviours, or that behavioural responses to a novel food present different underlying personality traits. The application of food neophobia to assess specific or generalized personality traits of dairy calves deserves further work.

## Introduction

Farm animals are exposed to various stressors, including diet changes, movement to a novel pen, and regrouping with unknown animals. Each of these examples involve exposure to novelty or challenging situations that are potentially stressful or fear-eliciting, and often result in individual differences in behavioural and physiological responses^1^. For example, in beef cattle that were challenged with restraint or isolation, individuals that were less reactive during weighing and handling (measured as degree of movement in a crush)^2,3^, and less reactive during a social separation test (measured as reduced activity)^4^, had improved feed intake and weight gains compared to more reactive individuals. Similar findings have been found in dairy calves, where more exploratory calves in a novel environment (measured as greater sniffing and licking) showed improved feed intake and performance around weaning^5^. Other behavioural characteristics appear to have implications for production; cows that were more fearful of humans (measured as avoidance of an unfamiliar human)^6,7^ or were more reactive to milking (measured as more steps and kicks during milking)^8^ produced less milk. Individual differences in behavioural responses during restraint and toward a human are also linked to physiological differences in metabolic responses to glucose and insulin challenge tests in beef cattle^9^. This evidence shows that cattle differ in how they respond to stressful events, including environmental and social novelty, which can have important impacts on the behaviour and physiology of individuals (reviewed by Neave *et al*.^10^).

Individual differences in behaviour that are consistent over time and across situations are termed ‘personality’ traits (or more commonly ‘animal temperament’^11^ in the farm animal literature), and these traits can relate to how individuals respond to novelty or challenging contexts^12^. The most commonly assessed personality trait in farm animals is ‘boldness’ (the propensity to take risks especially during novel situations^13^, but some literature uses an associated concept ‘fearfulness’, the negative emotional response to a real or perceived threat^1^) and ‘exploration’ (behaviour serving to gain information about the environment^14^); these are two of the so-called ‘Big Five’ animal personality traits described by Réale *et al*.^11^. Standardized tests of behavioural responses to novelty or challenging situations have been used in farm animals to infer these personality traits, including open field or novel environment, novel object, and novel human tests (reviewed by Forkman *et al*.^15^), and these traits exhibit some long-term consistency from young calf to adulthood^16^. However, the use of standardized tests in cattle may have some drawbacks. Most tests were initially developed for use in laboratory rodents^17^ and may lack ecological relevance for cattle^18^; for example, the open field test was originally developed for rodents (given that they are nocturnal and present thigmotaxis), but there is less reason to expect that cattle would be fearful of open environments (given that their natural behaviour places them in large open areas for grazing)^15^. Further, these standardized tests can be time consuming and may be impractical to perform on commercial farms. Therefore, there is a need to identify a practical test with ecological relevance for cattle, and determine if this can identify the ‘boldness’ and ‘exploratory’ personality traits identified using traditional tests.

Food neophobia is defined as the avoidance of and reluctance to taste unfamiliar foods^19^ (where neophobia refers to an aversion to or avoidance of novelty^18^); this phenomenon is commonly seen in ruminants^20^ and is thought to help animals avoid consumption of toxic plants^21^. For instance, dairy cows consumed very little carrots when exposed to them for the first time, but intake increased with increased length of exposure^22^. Costa *et al*.^23^ found that dairy calves reared in a complex social environment were quicker to approach and ate more of a novel food compared to calves reared individually; this behaviour was consistent within calves over 3 test days. The behaviours expressed during exposure to a novel food are thought to reflect fear or interest in the novel food, and a hesitancy to incorporate the novel food into the regular diet^24^. We hypothesize that these elements of food neophobia are related to boldness (e.g. latency to approach novel food) and exploratory (e.g. time spent inspecting and eating the novel food) personality traits that have previously been identified in other novelty tests in dairy calves^5,25,26^. Indeed, a novel food test has been employed in a number of other species as a means of measuring these personality traits (fish:^27,28^; birds: ^29–31^ primates:^32,33^). Thus, responses to a food neophobia test, assessing individual differences in behavioural responses to a novel food, may be related to other behavioural responses to novelty in cattle.

A few studies in ruminant farm animals have investigated the relationship between behavioural responses to food neophobia and other novel or stressful situations. Behaviour in an open-field test was related to reluctance to eat new foods in sheep, where individuals that were more reactive (measured as a higher number of bleats) ate less of the novel food^34^. However, a study in adult dairy cattle reported no association between behavioural responses toward a novel food (measured as time away from, sniffing or eating) and a novel human or object (measured as time away from or sniffing)^35^, but these findings were limited to dairy cattle in tie-stall housing, and no studies of this nature have been conducted in dairy calves. The food neophobia test may be particularly relevant to young dairy calves given that they are presented novel diets as part of standard farm management (e.g. during weaning from a liquid to solid feed diet, and from a grain-based to forage-based diet).

The objective of this study was to determine if individual differences in behavioural responses toward a novel food were comparable to behavioural responses toward an open-field, novel object and novel human in dairy calves, and if these behavioural responses reflected boldness and exploration personality traits across these different contexts. We hypothesized that (1) boldness in the novel object and novel human contexts (reflected in short latencies to approach and high time spent touching the object and human) will predict boldness in the novel food context (reflected in short latencies to approach and eat the novel food), and that (2) exploration in the open-field context (reflected in high time spent sniffing the environment and high activity) will predict exploration in the novel food context (reflected in high time spent inspecting and eating the novel food).

## Materials and methods

This experiment was conducted at The University of British Columbia’s (UBC) Dairy Education and Research Centre (Agassiz, British Columbia, Canada) between February and April 2015. Procedures were approved by the UBC Animal Ethics Committee (protocol #A15-0117) and were performed in accordance with the guidelines and regulations of the Canadian Council on Animal Care^36^.

### Animals and housing

Thirty-three Holstein calves were enrolled at (mean ± SD) 100 ± 11 d of age. At birth, calves were separated from their dam, moved to individual pens bedded with sawdust, and fed at least 4 L of colostrum (>50 g/L of IgG) by bottle within 6 h of birth. Calves were dehorned at 4 d ± 1 d of age using caustic paste (Dr. Naylor’s Dehorning Paste; H. W. Naylor Company, Inc., Morris, NY, USA) and sedation (Rompun, 2% Bayer Inc., Ontario, Canada; 0.25 mg/kg BW; following Vickers *et al*., 2005)^37^. These procedures in the first few days of life followed standard commercial farm practice. At d 7 ± 3 d, calves were moved to a sawdust-bedded group pen with 7 to 9 calves (one group of 7, one of 8, and two of 9 calves) per pen (7.0 m × 5.0 m); calves remained in these groups for the duration of the experiment. From d 7 to d 70 of age calves received 12 L/d of whole pasteurized milk. From d 70 of age milk was reduced by 20%/d for 5 d until calves were completely weaned at d 76 of age. Throughout the experiment, all calves had *ad libitum* access to water, grass hay (overall DM of 85.7%; chemical composition (% of DM): 18.8%, CP, 52.6% NDF, 32.0% ADF) and calf starter (Hi-Pro Medicated Calf Starter, Chilliwack, BC, Canada; overall DM of 89.3%; chemical composition (% of DM): 19.8% CP, 15.4% NDF, 9.4% ADF). A CF1000CS-Combi 19 automatic feeder (De Laval, Tumba, Sweden) measured individual intakes of calf starter. Sawdust was added once per week to the group pen.

### Test procedures

Individual behavioural responses to different novel or challenging situations were assessed using three traditional tests (open field, novel human, and a novel object tests^15^), and a novel food (food neophobia) test. In line with our previous work^5,25^, and other studies in dairy calves^26,38^, we anticipated these tests to identify boldness and exploratory personality traits. These tests were carried out individually in a test pen that was identical to the home pen except that access to the feeding equipment was prevented using plywood. The test pen was located adjacent to the home pens and in the same barn (maximum distance = 25 m). Before the tests began, calves were pre-exposed to the plastic buckets used in the food neophobia test; this also involved pre-exposure to the test pen. Calves were held as a group for 2 h in the empty test pen, returned to their home pen and 48 h later were again held as a group for 2 h in the test pen, only this time the test pen contained two empty white 20 L plastic buckets held in two bucket holders placed on the wall 3 m apart and 60 cm above the pen floor. After this initial pre-exposure period, calves were tested individually in the four tests in sequence (d 1: open field test, d 2: novel human test, d 3: novel object test and d 4: food neophobia test), with one test per day for four consecutive days between 1400 and 1700. For each test, the calf was guided gently from the home pen to the test pen by two trained individuals that were familiar to the calves. Calves entered the test pen in a randomized order each day.

In the open field test, calves entered the same test pen that they were previously held in as a group for the habituation period, except that the buckets were no longer present and the calf now experienced the test arena in isolation rather than in a group (thus, we refer to this test as an open field rather than a true novel environment test^39^); calves remained in the arena for 30 min to ensure familiarity of the pen for subsequent tests. In the novel human test, an unfamiliar female dressed in clean dark blue coveralls stood immobile at the centre of the pen. The person avoided eye contact with the calf by looking towards the feet of the calf and kept arms and hands inside the coverall pockets. In the novel object test, a black 140-L bucket was placed in the center of the pen. The durations of the novel human and object tests (10 and 15 min, respectively) were selected to balance logistical constraints with minimizing ceiling effects related to the calf’s latency to approach the human or object. The food neophobia test was performed following Costa *et al*.^23^. The test pen contained two white 20-L plastic buckets placed in each corner of the wall opposite to where the calf entered the pen. The bucket set up was identical to that in the pre-exposure period described above, except that one bucket contained 5 kg of a novel food (total mixed ration containing 49% DM, 26% corn silage, 15% grass silage, 10% alfalfa hay and 49% concentrated mix; chemical composition: 18.0% CP, 33.2% NDF, and 20.4% ADF) and the other bucket remained empty. The location of the food bucket (left or right) was randomized for each calf. The duration of the food neophobia test (30 min) followed a previous study in calves^23^ to ensure ample time for the calf to decide to approach and eat the novel food.

Behaviours during each test were observed using continuous video recording with two cameras (WV-CW504SP, Panasonic, Osaka, Japan) positioned 7 m above the test pen. A single observer, blind to the experimental objective, scored behaviours from video of each calf in each test using a detailed ethogram (supplementary material, Table S1; intra-observer reliability: ﻿κW > 0.85).

### Statistical analyses

All analyses were performed with SAS (version 9.3; SAS Inst. Inc., Cary, NC) using the calf as the experimental unit. All variables were summarized by calf for each test and were expressed as a percentage of test time. Data were scrutinized using the UNIVARIATE procedure and probability distribution plots in SAS.

We chose a correlational multivariate analysis approach to explore and extract common sets of behaviours across the different test contexts, followed by subjective interpretation of the meaning of these correlated sets of behaviours, guided by literature to assign labels to these sets of behaviours. Before analysis, variables were transformed to achieve normality if required (either log_10_ or square root transformations that achieved an adequate normal distribution). Frequencies of playing and bucking were too few to be meaningfully included in the analysis. A principal component analysis (PCA) was used to reduce correlated measures of the traditional tests (open field, novel human and novel object tests) into principal components (hereafter referred to as the ‘Novelty PCA’ for simplicity) which included 9 variables: latency to touch the human and object, time spent touching the human and object, time spent looking at the human and object, and time spent exploring, active and inactive. A second PCA was conducted using the measures in the food neophobia test (hereafter referred to as the ‘Food Neophobia PCA’) which included 6 variables: latency to approach and eat the novel food, intake of novel food, time spent eating, and time spent inspecting the food and empty buckets. For each PCA, analysis and reporting guidelines followed Budaev^40^. The correlation matrix was computed (supplementary material, Tables S2–S3) given that variables were measured on different scales and presented different variances (Table 1). Factors were retained if eigenvalues >1 and following scree plot examination. Factors were subjected to orthogonal (varimax) rotation, which is suggested for smaller sample sizes and to aid in interpretation of factor loadings. The variables used for analysis met the criteria of communality estimates (Novelty PCA > 0.59; Food Neophobia PCA > 0.68) and Kaiser–Meyer–Olkin measure of sampling adequacy (Novelty PCA = 0.52; Food Neophobia PCA = 0.51) required for conducting PCA. Individual scores on each of the PCA factors were extracted using the regression method.

**Table 1:** Behavioural responses (mean ± SD) of calves (n = 33) when tested in open field, novel human, novel object, and food neophobia tests.

Test / Behaviour	Mean	SD	Range
**Open field test**			
Exploration (% of test time)	42.6	15.4	16.4–73.4
Inactivity (% of test time)	42.0	14.9	11.8–65.3
Locomotor play (no.)	2.2	2.5	0–7
Bucking (no.)	1.5	2.0	0–8
Active (no. quadrants crossed)	36.3	15.6	7–66
**Novel human test**			
Latency to touch (s)	172	228	3–600
Looking at human (% of test time)	19.2	7.3	7.5–30.5
Inattentive (% of test time)	70.9	10.4	32.8–88.5
Touching human (% of test time)	10.0	11.8	0–56.8
Locomotor play (no.)	0.70	1.2	0–5
Bucking (no.)	0.18	0.53	0–2
**Novel object test**			
Latency to touch (s)	55	109	3–539
Looking at object (% of test time)	8.7	4.3	3.2–24.6
Inattentive (% of test time)	76.8	12.3	39.1–91.9
Touching object (% of test time)	14.0	11.0	0.3–51.3
Locomotor play (no.)	1.4	2.7	0–10
Bucking (no.)	0.6	1.2	0–6
**Food neophobia test**			
Intake of novel feed (g)	96.4	95.7	0–400
Latency to approach feed (s)	86	201	2–1193
Latency to eat feed (s)	334	530	17–1800
Time spent eating (% of test time)	6.2	5.5	0–20.5
Time in contact with feed bucket, excluding eating (% of test time)	3.9	6.1	0.2–34.8
Time in contact with empty bucket, including head in bucket (% of test time)	6.8	13.0	0–67.2

To determine whether factor scores derived from the traditional tests could be used to predict the factor scores derived from the food neophobia test, a linear regression was performed (PROC REG); scores on each factor from the Novelty PCA were the explanatory variables, and scores on each factor from the Food Neophobia PCA were the response variables. Group and age of calf were included as fixed effects, which were removed from the model using backwards elimination if *P* > 0.3. The R^2^ and *P*-value for each model, as well as the t-value and *P*-value for the explanatory factors in each model, are reported.

We chose to conduct two separate PCAs (one for the traditional tests, and one for the food neophobia test), followed by regression analysis of the individual factor scores extracted from the PCAs, to more directly compare how responses in the traditional tests are related to responses in the food neophobia test. An alternative statistical approach could have used a single PCA that included all recorded measures across all tests; this approach would have identified which behavioural measures from the food neophobia and traditional tests were most highly correlated. An additional approach could have conducted 4 PCAs, one for each test. We found that either of these approaches resulted in the same behaviours loading together on a factor of the PCA, meaning the same sets of behaviours were well correlated and resulted in similar conclusions regarding the association of behavioural measures in the food neophobia and traditional tests.

## Results

### Behavioural responses in the tests: principal component analysis

Behavioural responses of calves in the traditional and food neophobia tests (mean, SD and range) are presented in Table 1. Three principal components were retained in the Novelty PCA, accounting for 67.3% of the total variation in behavioural measures in the open field, novel human and novel object tests. The loadings for these factors are presented in Table 2. Factor 1 explained 31.7% of the total variation. For ease of understanding and to be consistent with literature terminology, the loadings on Factor 1 were inversed, resulting in high negative loadings for latency to touch the human and object, a high negative loading for looking at the human, and high positive loadings for touching the human and object. Calves with high scores on this factor were interpreted as ‘Bold’. Factor 2 explained 22.9% of the total variation and had high positive loadings for looking at the object and exploring the open field environment, and high negative loading for standing inactive. Calves with high scores on this factor were interpreted as ‘Exploratory’. Factor 3 explained 12.7% of the total variation with a high positive loading for activity. Calves with high scores on this factor were interpreted as ‘Active’.

**Table 2:** Loadings on the three factors extracted by principal component analysis (PCA) ^a^of behavioural measures recorded when calves ^b^were tested individually in open field, novel human and novel object tests.

Variable	Factor 1^c^	Factor 2	Factor 3	Communality estimate *h*^2^
Latency to touch human	−0.83	−0.13	−0.10	0.71
Latency to touch object	−0.67	0.35	0.16	0.59
Touching human	0.71	0.40	0.29	0.75
Touching object	0.77	−0.19	0.05	0.63
Looking at human	−0.67	−0.34	0.22	0.61
Looking at object	−0.19	0.73	0.17	0.59
Exploring	0.13	0.77	0.13	0.63
Active (no. quadrants crossed)	0.003	0.04	0.97	0.93
Inactive	−0.10	−0.78	0.01	0.61
Eigenvalues	2.9	2.1	1.1	
Variance explained (%)	31.7	22.9	12.7	
Interpretation (suggested label)^d^	Bold	Exploratory	Active	

Eigenvalues and proportions of total variation explained by each factor are reported. High loadings (considered ≥ ± 0.62) are used for interpretation.

^a^See supplementary material for the correlation matrix.

^b^Correlation matrix was calculated from n = 30 calves (of the original 33 calves; 3 had missing data for the open field test and were excluded from the matrix calculation).

^c^All loadings presented for Factor 1 have been inversed to aid in interpretation.

^d^Labels applied to each factor are subjective, based on interpretation of the meaning behind the correlated set of behaviours.

Three principal components were retained for the Food Neophobia PCA, accounting for 78.0% of the total variation in behavioural measures in the food neophobia test. The loadings for these factors are presented in Table 3. Factor 1 explained 38.6% of the total variation and had high positive loadings for intake of novel feed and time spent eating; calves with high scores on this factor were termed ‘Eating’. Factor 2 explained 22.2% of the total variation and had high positive loadings for time spent inspecting the feed and empty buckets; calves with high scores on this factor were termed ‘Inspecting’. Factor 3 explained 17.1% of the total variation and had high positive loadings for latency to approach and eat the novel food; calves with high scores on this factor were termed ‘Avoidance’.

**Table 3:** Loadings on the three factors extracted by principal component analysis (PCA) ^a^of behavioural measures recorded when calves ^b^were tested individually in a food neophobia test where calves were exposed to a novel mixed feed (TMR).

Variable	Factor 1	Factor 2	Factor 3	Communality estimate *h*^2^
Latency to eat	−0.05	−0.39	0.72	0.68
Latency to approach the feed	0.03	0.20	0.87	0.79
Intake of novel feed	0.94	−0.04	−0.07	0.89
Time spent eating	0.86	0.37	0.05	0.87
Time spent inspecting feed bucket	0.43	0.70	0.10	0.69
Time spent inspecting empty bucket	0.009	0.86	−0.11	0.77
Eigenvalues	2.3	1.3	1.0	
Variance explained (%)	38.6	22.2	17.1	
Interpretation (suggested label)^c^	Eating	Inspecting	Avoidance	

Eigenvalues and proportions of total variation explained by each factor are reported. High loadings (considered ≥ ± 0.62) are used for interpretation.

^a^See supplementary material for the correlation matrix.

^b^Correlation matrix was calculated from n = 31 calves (of the original 33 calves; 2 had missing data for the food neophobia test and thus were excluded from the matrix analysis).

^c^Labels applied to each factor are subjective, based on a rational interpretation of the meaning behind the correlated set of behaviours.

### Regression analyses

**Figure 1:**
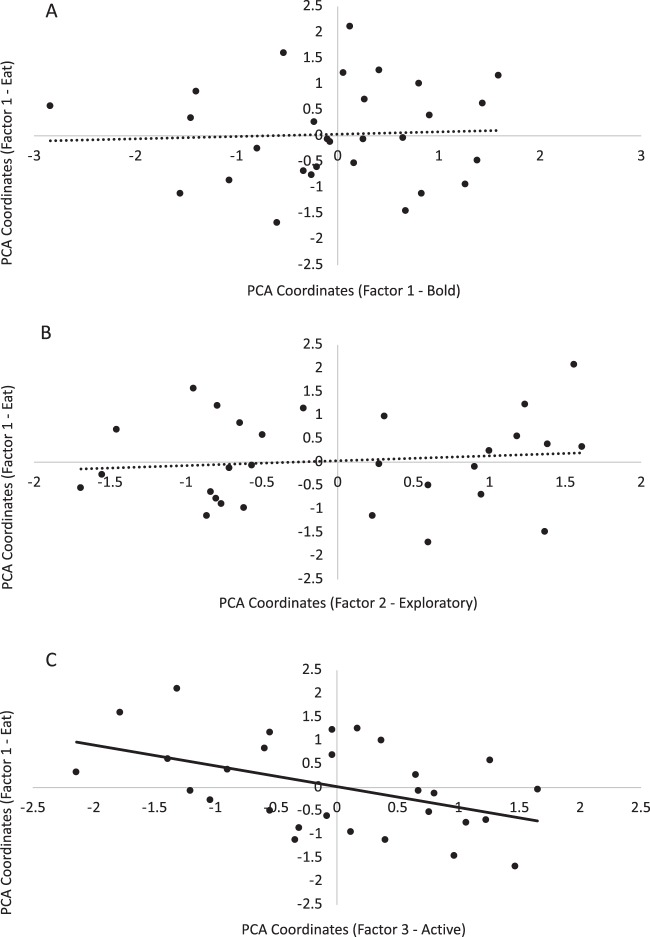
Distribution of individual calf scores on Factor 1 (‘Eating’) of the Food Neophobia PCA plotted against each factor of the Novelty PCA: (**A**) Factor 1 (‘Bold’), (**B**) Factor 2 (‘Exploratory’), and (**C**) Factor 3 (‘Active’). The linear regression trendline for each plot is presented (solid black line = significant regression, *P* < 0.05; dotted line = non-significant regression, *P* > 0.05).

**Figure 2:**
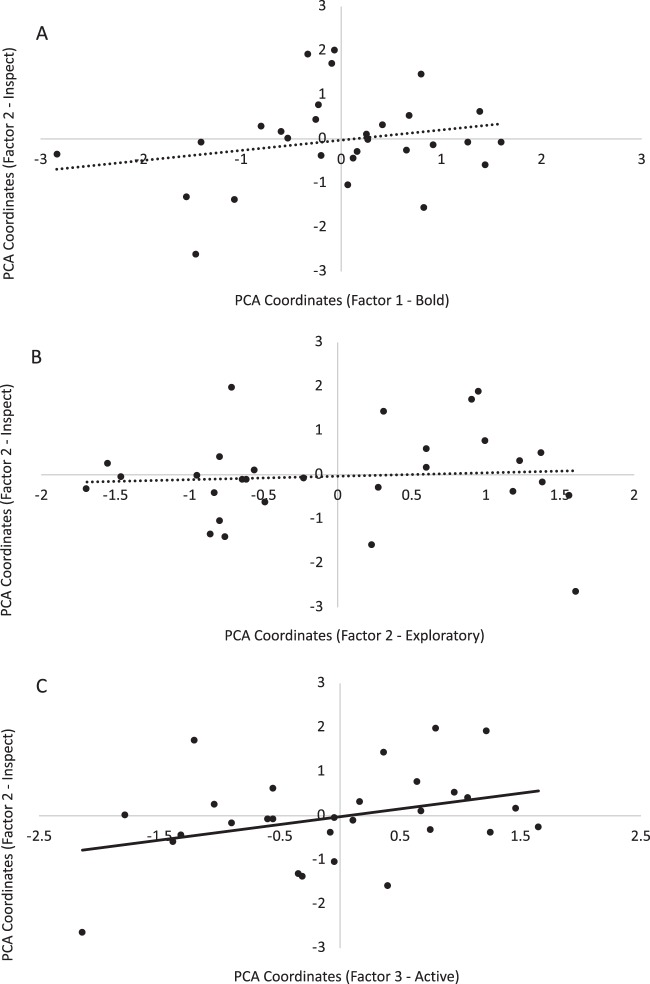
Distribution of individual calf scores on Factor 2 (‘Inspecting’) of the Food Neophobia PCA plotted against each factor of the Novelty PCA: (**A**) Factor 1 (‘Bold’), (**B**) Factor 2 (‘Exploratory’), and (**C**) Factor 3 (‘Active’). The linear regression trendline for each plot is presented (solid black line = significant regression, *P* < 0.05; dotted line = non-significant regression, *P* > 0.05).

**Figure 3:**
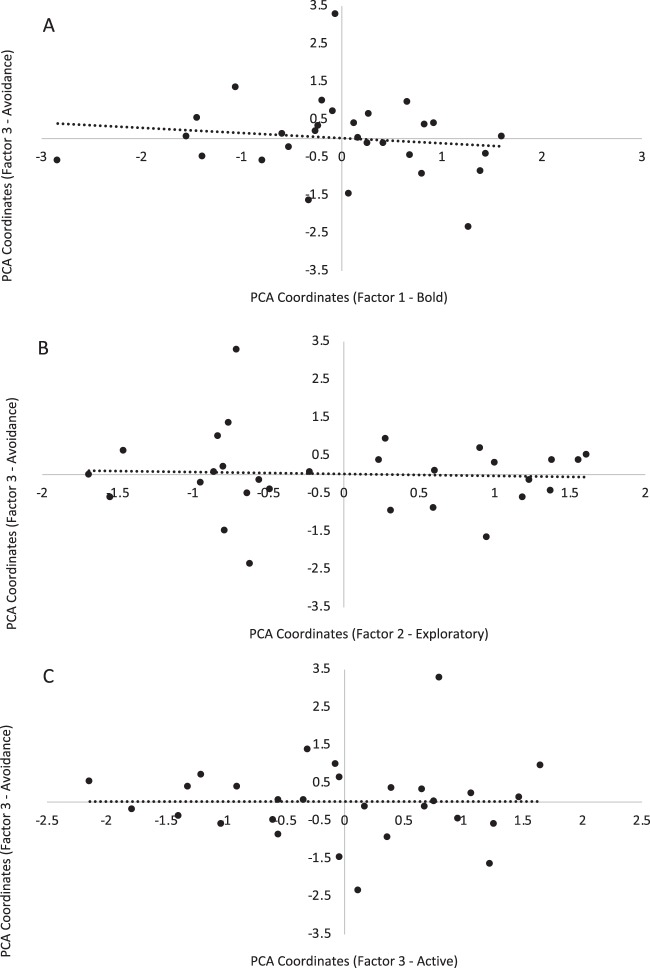
Distribution of individual calf scores on Factor 3 (‘Avoidance’) of the Food Neophobia PCA plotted against each factor of the Novelty PCA: (**A**) Factor 1 (‘Bold’), (**B**) Factor 2 (‘Exploratory’), and (**C**) Factor 3 (‘Active’). The linear regression trendline for each plot is presented (solid black line = significant regression, *P* < 0.05; dotted line = non-significant regression, *P* > 0.05).

The distribution of individual scores on each factor of the Novelty PCA (‘Bold’, ‘Exploratory’ and ‘Active’) are plotted against each factor of the Food Neophobia PCA (‘Eating’, Fig. 1; ‘Inspecting’, Fig. 2; and ‘Avoidance’, Fig. 3). Factor 1 (‘Eating’) of the Food Neophobia PCA could be explained by Factor 3 (‘Active’) of the Novelty PCA, but not by Factor 1 (‘Bold’) or Factor 2 (‘Exploratory’) (R^2^ = 0.35, *P* = 0.03; Table 4). Similarly, Factor 2 (‘Inspecting’) of the Food Neophobia PCA could be explained by Factor 3 (‘Active’) of the Novelty PCA, but not by Factor 1 (‘Bold’) or Factor 2 (‘Exploratory’) (R^2^ = 0.38, *P* = 0.04; Table 4). Finally, Factor 3 (‘Avoidance’) of the Food Neophobia PCA could not be explained by any of the Novelty PCA factors (R^2^ = 0.02, *P* = 0.91; Table 4). Thus, ‘bold’ and ‘exploratory’ traits identified in behavioural responses to the open field, novel object and novel human tests were not associated with the findings of the food neophobia test, but activity during the open field test reflected some aspects of behavioural responses during the food neophobia test.

**Table 4:** Parameter estimates describing the relationship between factor loadings derived from the Food Neophobia PCA (using variables from the food neophobia test) as the response variables and factor loadings derived from the Novelty PCA (using variables from the open field, novel human and novel object tests) as the explanatory variables.

Factors from Novelty PCA (explanatory variables)	Factors from Food Neophobia PCA (response variables)								
	Factor 1 (Eating)			Factor 2 (Inspecting)			Factor 3 (Avoidance)		
	Estimate ± SE	t-value	*P*-value	Estimate ± SE	t-value	*P*-value	Estimate ± SE	t-value	*P*-value
Factor 1 (Bold)	0.099 ± 0.16	0.63	0.53	0.25 ± 0.17	1.5	0.16	0.14 ± 0.20	0.67	0.51
Factor 2 (Exploratory)	0.16 ± 0.16	1.1	0.31	−0.065	0.37	0.71	−0.05 ± 0.20	0.25	0.80
Factor 3 (Active)	−0.42 ± 0.16	2.7	0.01	0.46 ± 0.17	2.7	0.01	−0.001 ± 0.20	0.01	0.99

## Discussion

This study investigated the relationship between behaviours expressed during exposure to a novel food versus exposure to an open field, novel object and novel human, and if these behavioural responses reflected boldness and exploratory personality traits. We found that behaviours in the novel object and novel human tests were well correlated, reflecting a boldness personality trait, and that behaviours in the open field test were not correlated with the novel object and novel human tests, reflecting exploration and activity personality traits. While correlated sets of behaviours in the food neophobia test did not predict scores for the boldness or exploratory personality traits measured in the traditional tests, some behaviours in the food neophobia test did predict scores for the activity personality trait. This result indicates that when calves are faced with a novel food, their behavioural responses may not reflect boldness or exploratory traits, and may instead reflect a different personality trait or they may be context-specific. We discuss explanations for these findings in light of the limitations of our study.

We observed considerable variation among calves in response to the novel food, including latency to approach (range: 2 s to 20 min), time spent inspecting (range: 0.2 to 35% of the 30 min testing period), and food intake (range: 0 to 400 g). This variability reflects individual differences in responses to a novel food and aligns with the findings of other studies showing reluctance to eat a novel food in young^23^ and mature dairy cattle^22,35^. These behavioural differences in the food neophobia test likely stem from individual differences in both fear and interest in novelty. Exposure to novel foods is thought to involve two types of neophobia: fear or interest in the novel food, and hesitancy to incorporate the novel food into the regular diet^18,24^. We frame the discussion of our results around how these elements of food neophobia relate to behavioural responses toward novelty or challenge in other contexts.

The appearance of the novel food may elicit behavioural responses that reflect a type of object neophobia (aversion or avoidance of novelty)^18^. For instance, responses toward novel objects and novel foods were well correlated in deer^41^ and sparrows^42^; thus we expected that the initial response of calves toward the novel food (measured as latency to approach and eat the novel food) would predict scores for the boldness personality trait derived from the novel object and novel human tests. We found that latencies to approach and eat the novel food were correlated, forming their own factor following data-reduction in the PCA (which we interpreted as ‘avoidance’), but this set of behaviours did not predict boldness during novel object and novel human tests. Herskin *et al*.^35^ also reported no consistency in behavioural responses between novel food, novel object and novel human tests in mature cattle. These authors argued that the novel food elicited a stronger behavioural response compared to presentation of their usual food, and that the novel object or human in their study may not have been fear inducing. Herskin *et al*.^35^ housed cows in tie-stalls, which likely limited behavioural expression when exposed to these stimuli. Other work has reported a negative correlation between latencies to approach a novel food and a novel object^43^. Together this work suggests that these ‘avoidance’ behaviours in the food neophobia test may reflect an underlying trait that is unrelated to other behaviours expressed during the food neophobia test, and unrelated to boldness measured in other novel contexts.

The initial presentation of novel foods may also elicit interest, often measured as exploratory behaviour (i.e. neophilia, the exploration of novelty)^44^. Individual variation in neophilia has most often been reported in birds, especially juveniles, in the context of new foraging resources^45^. Furthermore, sampling and acceptance of a new food is necessary for incorporating a novel food into the regular diet, which is argued to be a distinct process separate from initial fear of approaching and investigating the novel food^24^. Both elements of food neophobia describe some level of exploratory behaviour, which we measured as time spent investigating and eating the novel food, and total intake of the novel food. We expected that these sets of behaviours in the food neophobia test would be correlated, and would predict scores for the exploratory personality trait derived from the open field test; however, our results did not support this prediction. Time spent inspecting the food and empty buckets were correlated on their own factor dimension in the PCA (which we interpreted as ‘Inspecting’), while time spent eating and total intake of food were a separate set of correlated behaviours on a third dimension of the PCA (which we interpreted as ‘Eating’). Scores on these factor dimensions were, however, associated with scores on the ‘Active’ personality trait, where ‘Inspecting’ behaviours presented a positive association and ‘Eating’ behaviours presented a negative association. This suggests that eating more of a novel food is associated with decreased activity in a different novel context. In contrast, Villalba *et al*.^34^ found that the number of grid lines crossed during a novel environment test (the same measure of activity used in the current study) did not correlate with novel feed intake in lambs. However, lambs that bleated more in the novel environment test consumed less of the novel food, which the authors suggested reflected the influence of sociability on reluctance to ingest a novel food. Other work showed that increased activity of cattle in a test arena was associated with greater reactivity to social isolation (a similar design to that of the current study)^4^. These studies suggest that increased vocalizations and activity may reflect a negative behavioural response to social isolation rather than toward novelty per se; thus the ‘Active’ personality trait identified in this study may reflect dairy calves’ response to social isolation, which may be influential in the sampling and acceptance of novel foods. Given that each of our tests were conducted in isolation from the group, we encourage future work to explore how food neophobia is expressed in a group setting where more sociable calves may be more attentive to social cues that provide information about the feeding environment and encourage consumption of different feeds.

Our study has some limitations that should be considered in future work. For the food neophobia test to be considered a personality test (either reflecting overlapping traits identified with the traditional tests, or reflecting distinct underlying personality traits), the test must meet the criteria of consistency across time and across contexts, as has been established for the traditional tests^16^. These tests have also been validated in a pharmacological study where an anxiolytic increased activity and time spent in contact with a novel object in dairy calves^46^. Following the guidelines of Carter *et al*.^39^, multiple measures that are expected to measure multiple traits should be employed to determine repeatability, and convergent and discriminant validity of the food neophobia test in calves. One previous study did report that the food neophobia test that we employed was consistent across repeated tests within individuals^23^.

In addition, our findings are limited to the food neophobia test methodology we used. Although all tests were performed in social isolation, the presentation of the novel food in combination with this non-social context may have resulted in behavioural expression unique to this situation. Behavioural responses in our food neophobia test could also have been affected by aspects of the food unrelated to novelty (such as texture, flavor or food presence in this particular context), or how hungry or food-motivated the calf was at the time of testing; this limitation could be addressed by measuring behavioural responses toward both a familiar food and unfamiliar food. We also suggest to directly investigate reluctance to incorporate the novel food into the individual’s regular diet, by performing repeated presentations of the food and measuring intake over time.

Finally, it is possible that standard commercial practices of maternal separation, individual housing and dehorning that occur early in life may affect how calves respond to stressful situations, including those that are novel, later in life. There is evidence for modulation of personality traits with early-life experiences in other species (see references in Langenhof and Komdeur^47^); this presents an important line of research in dairy cattle given the human interventions experienced from birth.

Overall, the identification of personality traits of dairy cattle is an important field of research with animal welfare, productivity and economic benefits. Personality traits in beef and dairy cattle have been associated with a number of performance measures including feeding behaviour^2,3,48^, weight gain^49,50^, fertility^51,52^, and milk production^8,53^ (also see reviews by Neave *et al*.^10^ and Haskell *et al*.^54^). However, common tests of responses to novelty or challenge in dairy cattle (open field, novel object or novel human tests) are often impractical to conduct on farms; the food neophobia test has potential to be an ecologically relevant test that can be easily implemented on-farm. Food neophobia tests may also indicate behavioural flexibility or adaptability of livestock managed under different housing environments (dairy calves:^23^; pigs:^55^); this information would be useful to identify animals that are likely to adapt to changes in feed or other environmental changes that occur on farms. Further work is required to understand the underlying personality traits measured with a food neophobia test; the results of this study can be used as a starting point to investigate relationships with behaviours measured in traditional novelty or challenge tests applied to livestock.

## Conclusions

A food neophobia test for behavioural responses to a novel food resulted in several sets of correlated behaviours, reflecting eating, inspecting and avoidance behaviours. However, these sets of behaviours did not predict boldness or exploratory personality traits derived from open field, novel object and novel human tests. Eating and inspecting behaviours were associated with the scores for the active personality trait. These results suggest that the food neophobia test in our study resulted in context-specific behaviours, or that behavioural responses to a novel food present different underlying personality traits. We recommend further work to investigate the application of food neophobia to assess specific or generalized personality traits of dairy calves.

## Supplementary information

Supplementary Material.

## Data Availability

Data collected and analyzed with SAS code are available electronically in the Mendeley data repository at http://dx.doi.org/10.17632/2twtspv9wv.2.
