# Supplementary material for: Use of a food neophobia test to characterize personality traits of dairy calves
Source: Sci Rep. 2020 Apr 28;10:7111. doi: 10.1038/s41598-020-63930-8 (PMC7188825; doi:10.1038/s41598-020-63930-8)
Supplement: Supplementary file 1 — Supplementary Material. [file 41598_2020_63930_MOESM1_ESM.docx]

**Supplementary Material for:**

**“Use of a food neophobia test to characterize personality traits of dairy calves”**

**J.H.C. Costa, H.W. Neave, D.M. Weary, and M.A.G. von Keyserlingk**

**Table S1.** Ethogram of behaviors scored during each of the personality tests. Calves (n = 33) were tested individually in open field, novel human, novel object and food neophobia tests at 100 ± 11 d of age. Calves remained in each of the tests for 30, 10, 15 and 30 min, respectively.

| Test / Behavior | | Description |  |
| --- | --- | --- | --- |
| All tests ^1^ | |  |  |
|  | Locomotor Play | Jumping: both forelegs off the ground and extended forwards (number of events)  Running: calf trotting (2 beats) or galloping (3 beats) across or around the enclosure (number of events) |  |
|  | Bucking | Both hind legs are off the ground and extended backwards (number of events) |  |
|  | Resting | Time spent lying down with underside or side of body in full contact with flooring substrate |  |
| Open field test ^2^ | |  |  |
|  | Exploration | Time spent with muzzle or tongue in contact with either walls or flooring substrate while moving or stationary |  |
|  | Active | Total number of squares crossed with all four feet (test arena divided into 4 equal quadrants) |  |
|  | Inactive | Time spent standing still without sniffing or licking of walls or floor |  |
| Novel human and novel object tests ^2^ | |  |  |
|  | Latency to Touch | Time until moment calf touches the human or object (muzzle within 5 cm, oriented toward the human or object) |  |
|  | Attentive | Time spent with head oriented toward human or object. Far: more than one body length away. Close: less than one body length away. |  |
|  | Inattentive | Time spent with head oriented away from human or object and engaged in a behavior other than those in ethogram |  |
|  | Touching | Time spent with muzzle in contact with human or object (muzzle within 5 cm, oriented toward the human or object) |  |
|  | Object play | Butting (head in contact with) human or object, or ‘mock butt’ where head is oriented downward and toward but not in contact with human or object |  |
| Food neophobia test ^3^ | |  |  |
|  | Latency to approach food bucket | Time until moment calf touches full food bucket |  |
|  | Latency to eat | Time until moment calf places head inside of food bucket to eat, ending with collection or chewing of food |  |
|  | Eating | Time spent collecting (head inside of food bucket) or chewing food |  |
|  | Head in empty bucket | Time spent with head inside of empty food bucket |  |
|  | Touching | Time spent with any body part in contact with full or empty food bucket, including sniffing or licking bucket |  |

^1^ These measures were excluded from principal component analyses due to low occurrences

^2^ These measures were included in the Novelty principal component analysis

^3^ These measures were included in the Food Neophobia principal component analysis

**Table S2.** Correlation matrix used in Principal Component Analysis of the traditional tests (i.e. Novelty PCA).

| Correlations | | | | | | | | | |
| --- | --- | --- | --- | --- | --- | --- | --- | --- | --- |
|  | IndHA_Lat_log10 | IndHA_T_sqrt | IndHA_look | IndNO_Lat_log10 | IndNO_T_log10 | IndNO_look_sqrt | IndNE_TotalQ | IndNE_I | IndNE_TotalLick |
| IndHA_Lat_log10 | 1.00000 | -0.58273 | 0.62025 | 0.36015 | -0.50901 | -0.01249 | -0.11566 | 0.09521 | -0.10184 |
| IndHA_T_sqrt | -0.58273 | 1.00000 | -0.50831 | -0.28423 | 0.44349 | 0.20443 | 0.21530 | -0.20939 | 0.39295 |
| IndHA_look | 0.62025 | -0.50831 | 1.00000 | 0.18981 | -0.24635 | -0.08903 | 0.13299 | 0.29690 | -0.13394 |
| IndNO_Lat_log10 | 0.36015 | -0.28423 | 0.18981 | 1.00000 | -0.48983 | 0.30840 | 0.09751 | -0.07714 | 0.10687 |
| IndNO_T_log10 | -0.50901 | 0.44349 | -0.24635 | -0.48983 | 1.00000 | -0.30373 | -0.01183 | 0.02560 | 0.10201 |
| IndNO_look_sqrt | -0.01249 | 0.20443 | -0.08903 | 0.30840 | -0.30373 | 1.00000 | -0.14794 | -0.33075 | 0.32810 |
| IndNE_TotalQ | -0.11566 | 0.21530 | 0.13299 | 0.09751 | -0.01183 | -0.14794 | 1.00000 | 0.00633 | 0.00682 |
| IndNE_I | 0.09521 | -0.20939 | 0.29690 | -0.07714 | 0.02560 | -0.33075 | 0.00633 | 1.00000 | -0.60599 |
| IndNE_TotalLick | -0.10184 | 0.39295 | -0.13394 | 0.10687 | 0.10201 | 0.32810 | 0.00682 | -0.60599 | 1.00000 |

**Table S3.** Correlation matrix used in Principal Component Analysis of the food neophobia test (i.e. Food Neophobia PCA).

| Correlations | | | | | | |
| --- | --- | --- | --- | --- | --- | --- |
|  | IndNEO_Intake_sqrt | IndNEO_LatEat_Per_log10 | IndNEO_LatApproach_Per_log10 | IndNEO_E_sqrt | IndNEO_AF_log10 | IndNEO_Totalempty_log10 |
| IndNEO_Intake_sqrt | 1.00000 | -0.03719 | -0.04171 | 0.70550 | 0.31374 | 0.09369 |
| IndNEO_LatEat_Per_log10 | -0.03719 | 1.00000 | 0.29879 | -0.20576 | -0.14241 | -0.24844 |
| IndNEO_LatApproach_Per_log10 | -0.04171 | 0.29879 | 1.00000 | 0.18257 | 0.11946 | -0.00743 |
| IndNEO_E_sqrt | 0.70550 | -0.20576 | 0.18257 | 1.00000 | 0.54868 | 0.28777 |
| IndNEO_AF_log10 | 0.31374 | -0.14241 | 0.11946 | 0.54868 | 1.00000 | 0.44060 |
| IndNEO_Totalempty_log10 | 0.09369 | -0.24844 | -0.00743 | 0.28777 | 0.44060 | 1.00000 |
